# 36 h fasting of young men influences adipose tissue DNA methylation of *LEP* and *ADIPOQ* in a birth weight-dependent manner

**DOI:** 10.1186/s13148-017-0340-8

**Published:** 2017-04-21

**Authors:** Line Hjort, Sine W. Jørgensen, Linn Gillberg, Elin Hall, Charlotte Brøns, Jan Frystyk, Allan A. Vaag, Charlotte Ling

**Affiliations:** 1 Department of Endocrinology (Diabetes and Metabolism), Rigshospitalet, section 7652, Tagensvej 20, DK-2200 Copenhagen N, Denmark; 2 Faculty of Health and Medical Sciences, University of Copenhagen, Copenhagen, Denmark; 3 The Danish Diabetes Academy, Odense, Denmark; 4 Steno Diabetes Center, Gentofte, Denmark; 5 Epigenetics and Diabetes and Islet Cell Exocytosis, Department of Clinical Sciences, Lund University Diabetes Centre, Lund University, CRC, Jan Waldentröms gata 35, SE-20502 Malmö, Sweden; 6 Institute of Clinical Medicine, University of Aarhus, Aarhus, Denmark; 7 AstraZeneca, Mölndal, Sweden

**Keywords:** Epigenetics, Fasting, Type 2 diabetes, Low birth weight, Adipose tissue, Leptin, Adiponectin

## Abstract

**Background:**

Subjects born with low birth weight (LBW) display a more energy-conserving response to fasting compared with normal birth weight (NBW) subjects. However, the molecular mechanisms explaining these metabolic differences remain unknown. Environmental influences may dynamically affect epigenetic marks, also in postnatal life. Here, we aimed to study the effects of short-term fasting on leptin (*LEP*) and adiponectin (*ADIPOQ*) DNA methylation and gene expression in subcutaneous adipose tissue (SAT) from subjects with LBW and NBW.

**Methods:**

Twenty-one young LBW men and 18 matched NBW controls were studied during 36 h fasting. Eight subjects from each group completed a control study (overnight fast). We analyzed SAT *LEP* and *ADIPOQ* methylation (Epityper MassARRAY), gene expression (q-PCR), and adipokine plasma levels.

**Results:**

After overnight fast (control study), *LEP* and *ADIPOQ* DNA methylation levels were higher in LBW compared to those in NBW subjects (*p* ≤ 0.03) and increased with 36 h fasting in NBW subjects only (*p* ≤ 0.06). Both *LEP* and *ADIPOQ* methylation levels were positively associated with total body fat percentage (*p* ≤ 0.05). Plasma leptin levels were higher in LBW versus NBW subjects after overnight fasting (*p* = 0.04) and decreased more than threefold in both groups after 36 h fasting (*p* ≤ 0.0001).

**Conclusions:**

This is the first study to demonstrate that fasting induces changes in DNA methylation. This was shown in *LEP* and *ADIPOQ* promoters in SAT among NBW but not LBW subjects. The altered epigenetic flexibility in LBW subjects might contribute to their differential response to fasting, adipokine levels, and increased risk of metabolic disease.

**Electronic supplementary material:**

The online version of this article (doi:10.1186/s13148-017-0340-8) contains supplementary material, which is available to authorized users.

## Background

DNA methylation is the most studied epigenetic feature and was initially considered to be mitotically stable. However, today we experience an increasing understanding of environmental regulation of DNA methylation in adult life, and recent studies have shown that ageing [1–3], exercise [4, 5], and dietary factors [6–9] can alter site-specific DNA methylation in humans, across different tissues. Epigenetic modifications have further been associated with differential gene expression and altered metabolism in key diabetic tissues, including adipose tissue [4, 10–12].

Adipose tissue regulates energy homeostasis by storing lipids and secreting adipokines, and dysregulation of adipokine secretion has been shown to be directly involved in the pathophysiology of the metabolic syndrome [13, 14]. The most important adipokines include leptin, a key hormone regulating satiety that show a high correlation between adipose tissue mass and plasma levels [15] and adiponectin, an important regulator of glucose and lipid metabolism, which, in spite of its exclusive secretion from adipose tissue, shows a negative correlation with visceral adiposity [16].

Type 2 diabetes (T2D) and obesity are complex and multi-factorial diseases with an etiology dependent upon both genetic and environmental factors, where also the prenatal environment may play an important role [17]. The establishment of epigenetic modifications during fetal development is dependent on maternal lifestyle [18], placental function, and nutrient supply [19] and may link an adverse prenatal environment with higher risk of developing metabolic diseases in postnatal life [20, 21]. Being born with low birth weight (LBW) has been confirmed in several human studies to be associated with increased risk of developing insulin resistance and T2D in adult life [22–24]. In this regard, several studies have reported alterations of epigenetic patterns and plasticity in human tissues that are relevant to metabolic diseases in subjects born at term with a LBW compared to normal birth weight (NBW) subjects [6, 8, 9, 25].

LBW individuals have been characterized with increased total [23] and abdominal [6] fat mass in early adulthood compared to NBW individuals. Previously, we have found that when exposed to high-fat overfeeding, young LBW men respond with less increase in fasting plasma leptin (p-leptin) levels compared to NBW subjects [26]. This suggests an impaired regulation of leptin secretion and/or expression among LBW subjects. Additionally, we recently showed that LBW subjects display a more energy-conserving response to fasting compared with NBW subjects [27]. However, whether adipokine dysregulation is involved in development of metabolic diseases among LBW subjects and, importantly by which mechanisms, remains to be elucidated.

To our knowledge, the effects of fasting on DNA methylation have not been examined previously. In the present study, we investigated whether DNA methylation and expression of *LEP* and *ADIPOQ* were affected by 36 h fasting, in subcutaneous adipose tissue (SAT) from young, healthy NBW and LBW men.

## Methods

### Study design

As previously published [27], 21 LBW and 18 NBW subjects were recruited from the Danish National Birth Registry according to LBW (birth weight ≤10th percentile) or NBW (50th percentile ≤ birth weight ≤ 75th percentile). All participants were born at term (week 39–41) and were matched as healthy, non-diabetic, young males, with no history of diabetes in two generations and with a BMI <30 kg/m^2^. All participants were subjected to 36 h fasting, and after 8–16 weeks, 7 LBW and 6 NBW subjects were examined again during a control study, conducted through a 36-h period where the subjects received a diet equal to the standardization meals, and where biopsies were excised after an overnight (12 h) fast. Furthermore, 1 LBW and 2 NBW subjects returned after 3 days of standardization by control diet, only to participate by collection of overnight fasting samples (12 h) and tissue biopsies as described below. Thus, control study samples were obtained after an overnight fast from a total of 8 LBW and 8 NBW subjects who all also participated in the 36-h fasting study.

For 72 h prior to the fasting or control study interventions, the participants received a control diet of precooked meals to achieve standardization of energy intake (10 MJ per day, 50% carbohydrate, 35% fat, 15% protein). In addition the participants were not allowed to perform exercise or consume alcohol or soft drinks in these 3 days. During both the fasting and control study, the participants were allowed ad libitum water.

### Clinical examinations

**Fig. 1:**
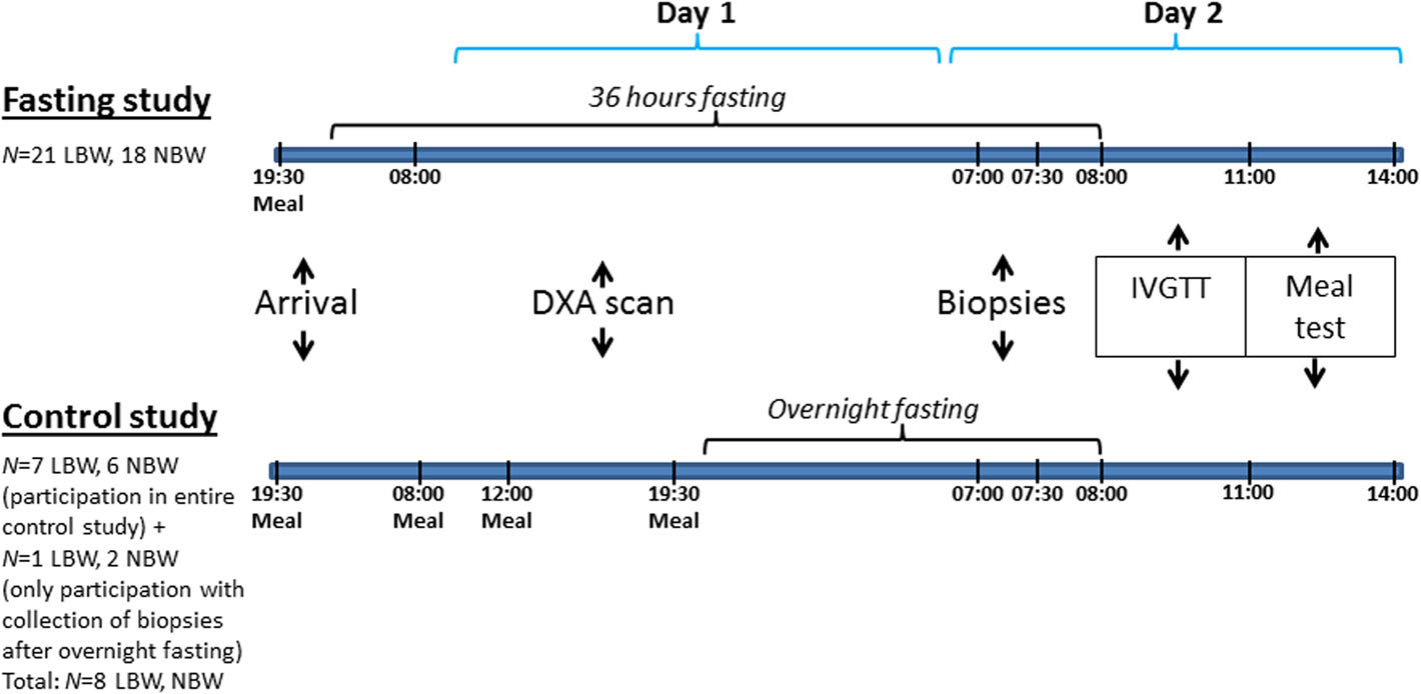
Study design. Overview of the study activities and time periods of fasting shown in both the fasting study and the control study. 21 LBW and 18 NBW men participated in the 36-h fasting study intervention. A subset of 7 LBW and 6 NBW men from the 36-h fasting study also participated in the control study intervention. Further 1 LBW and 2 NBW men also participated in the control study only with biopsy collections. Abbreviations: *LBW* low birth weight, *NBW* normal birth weight, *DXA scan* dual-energy X-ray absorptiometry scan, *IVGTT* intravenous glucose tolerance test

The fasting intervention study and the control study were performed with identical study settings and activities, carried out over 3 days (Fig. 1). The participants arrived at Steno Diabetes Center at 7.30 p.m., where they received a meal. In the fasting study, finishing of the meal marked the beginning of the fasting period. The next day (day 1), a catheter was placed in the subjects’ left arm and blood sampling began at 8.00 a.m. During day 1, anthropometric measurements of weight, height, BMI, and waist-hip ratio were obtained and dual-energy X-ray absorptiometry (DXA) scanning (Hologic Discovery QDR Series) was performed for determination of body composition. Furthermore, the participants had two periods of light exercise to avoid inactivity. On day 2, after 36 h fasting, between 7.00 and 7.30 a.m., abdominal SAT biopsies were obtained using a Bergström needle under suction and under local anesthesia by Xylocain (AstraZeneca). Immediately biopsies were frozen in liquid nitrogen and stored at −80 °C. At 8.00 a.m., an Intravenous Glucose Tolerance Test (IVGTT) was initiated (ending the fasting period), and at 11.00 a.m., the participant received a standardized test meal, which should be consumed within 15 min.

In brief, LBW subjects were characterized by a shorter height (*p* = 0.01) and lower lean body mass (*p* < 0.03), but with similar fat mass, total body fat percentage (BF%), BMI, and metabolic markers as NBW subjects. In both groups, the fasting intervention increased free fatty acid levels and reduced the clearance of p-glucose during the IVGTT, most likely due to development of peripheral insulin resistance as a consequence of fasting. Furthermore, we found that LBW subjects had a more pronounced decrease in s-insulin and triglyceride levels and exhibited a decrease of the total rate of energy expenditure during fasting which increased among NBW subjects. These data have previously been reported in more detail [27] and are summarized in Table 1.

**Table 1:** Clinical characteristics of the study participants

	LBW	NBW	*p* value
	*N = 21*	*N = 18*	
At baseline			
Birth weight (g)	2811 (205)	3643 (185)	*<* ***0*** *.* ***0001***
Gestational age (weeks)	40 (0.9)	39.9 (0.7)	0.83
Age (years)	24.8 (1.4)	24.6 (1.2)	0.65
Height (cm)	180.7 (5.7)	185.3 (5.3)	***0*** *.* ***01***
Weight (kg)	73.5 (10)	78.5 (10.7)	0.14
BMI (kg/m^2^)	22.5 (2.6)	22.9 (3.2)	0.65
W/H ratio	0.9 (0.07)	0.89 (0.05)	0.76
Subtotal fat (kg)	12.2 (5.2)	12.4 (5.4)	0.90
Subtotal fat (%)	17.1 (5.2)	16.3 (5.1)	0.61
Subtotal lean (kg)	54.6 (5.8)	59.2 (6.7)	***0*** *.* ***03***
HgbA1c (%)	5.1 (0.3)	5.1 (0.2)	0.87
Total cholesterol (mmol/L)	4.0 (0.6)	4.3 (0.7)	0.20
HDL (mmol/L)	1.11 (0.23)	1.13 (0.27)	0.80
LDL (mmol/L)	2.5 (0.6)	2.7 (0.6)	0.28
Triglyceride (mmol/L)	0.95 (0.24)	1.04 (0.34)	0.35
P-glucose (mmol/L)	4.7 (0.3)	4.8 (0.4)	0.41
S-insulin (pmol/L)	26 (10)	23 (14)	0.55
S-C-peptide (pmol/L)	441 (123)	441 (156)	0.98
Response during fasting			
Energy expenditure (kJ/24 h)	−46.1 (−2.8%)	+85.6 (+5.1%)	***0*** *.* ***02***
S-insulin (pmol/L)	−16.6 (−60%)	−11.1 (−42%)	***0*** *.* ***05***
Triglyceride (mmol/L)	−0.18 (−19.0%)	−0.10 (−9.9%)	***0*** *.* ***02***

Data are means (SD). *p* values <0.05 are bold. Lipids, glucose and insulin/C-peptide were measured at 8.00 AM on day 1, after 12 h of fasting (in the fasting study). HgbA1c was measured before the standardization period. Change in energy expenditure was calculated from after 12 h fasting until 31 h fasting. Changes in insulin and triglyceride during fasting were calculated from after 12 h fasting until 34 and 36 h fasting, respectively. These results have previously been reported [27]

*BMI* body mass index, *W/H ratio* waist-hip ratio, *Subtotal* without the head, *Lean* without fat and bone

### Intravenous glucose tolerance test

For evaluation of in vivo beta cell function, an intravenous glucose tolerance test (IVGTT) was conducted. A dose of 0.3 g glucose/kg body weight was infused and caused a first-phase insulin response (FPIR), which peaked within 2–5 min and lasted approximately 10 min. Twenty minutes after administration of the glucose dose, an IV insulin bolus of 0.02 IU insulin/kg (Actrapid®, Novo Nordisk) was infused. The IVGTT assessment and the calculations of insulin sensitivity have been described in detail previously [27].

### Blood sampling and biomarker analysis

All plasma samples were immediately distributed into tubes, placed on ice, and centrifuged at 3000 rpm for 15 min (Eppendorf Centrifuge 5810R, Eppendorf AG, Hamburg, Germany). Plasma and serum were obtained and stored at −80 °C for later analysis. Blood sampling for plasma leptin and adiponectin (p-leptin, p-adiponectin) was performed immediately after 12 and 36 h of both the fasting and control study, 3 h after initiation of the IVGTT, and 3 h after the meal test (Fig. 1).

Plasma adiponectin and leptin were determined by validated, in-house, monoclonal immunoassays, based on commercial reagents from Bio-Techne (Abingdon, UK). The assays were performed without any pre-treatment of samples prior to assay apart from appropriate dilution. Adiponectin was determined using MAB 10651 for coating and BAM 1065 for detection, leptin was determined using MAB 398 for coating and BAM 398 for detection. Recombinant proteins served as assay calibrators. All samples were assayed in duplicates, with intra-assay coefficients of variation (CV) of unknown samples <5% and inter-assay CVs of control samples <10% [28, 29]. All concentrations below the lower limit of detection (the non-specific binding control plus three standard deviations (SD)) were arbitrarily set at 0.1 μg/L for p-leptin, and 0.1 mg/L for p-adiponectin (total).

### DNA methylation analyses

**Fig. 2:**
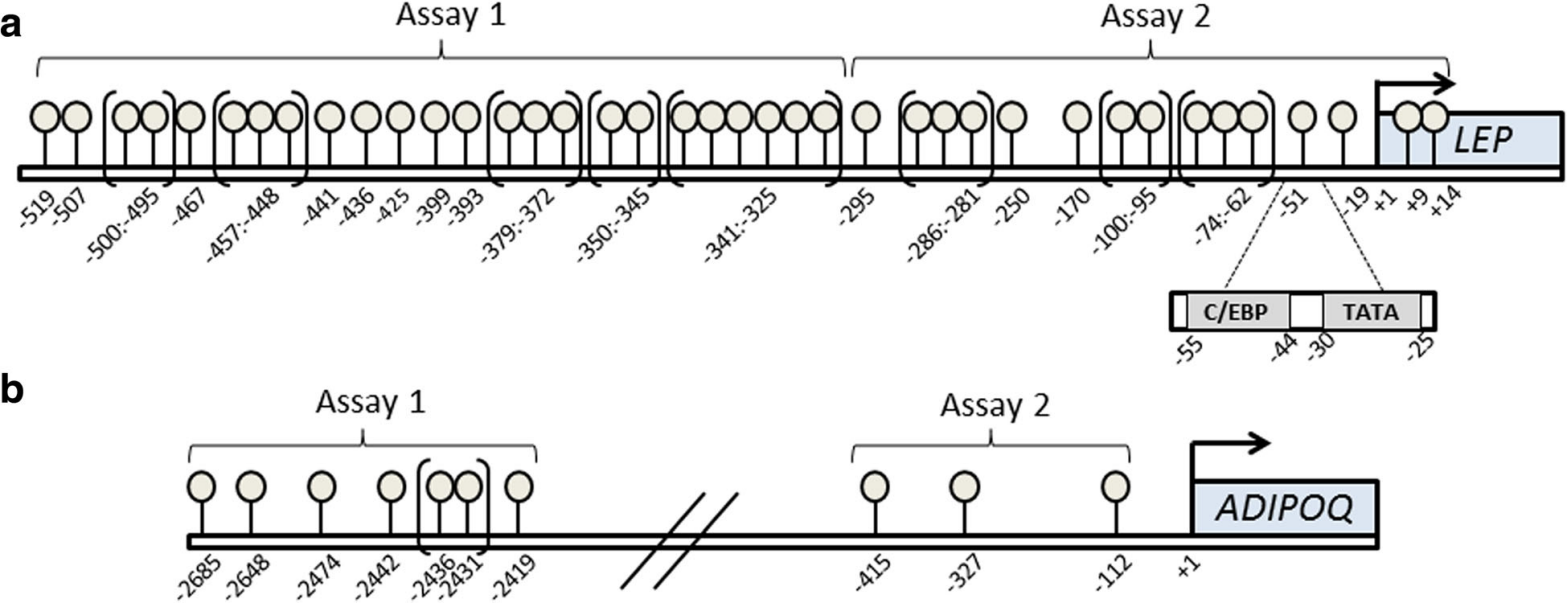
Assay design. Overview of successfully analyzed CpG sites in *LEP* (**a**) and *ADIPOQ* (**b**) by EpiTYPER. For *LEP*, two EpiTYPER assays were designed to cover the proximal *LEP* promoter region (*Assay 1* and *Assay 2*). For *ADIPOQ*, one assay was designed to cover the distal enhancer region (*Assay 1*) and one assay to cover a proximal promoter region (*Assay 2*). Due to base-specific cleavage, several CpG sites were analyzed as units, as indicated with *brackets*. The binding sites for transcription factor C/EBP (CCAAT-enhancer-binding protein) (−55 to −44) and the TATA box (−30 to −25) is marked on the *LEP* promoter (**a**)

Genomic DNA was extracted from SAT biopsies using the QIAamp DNA Mini Kit (Qiagen, Valencia, CA, USA). To quantify DNA methylation of *LEP* and *ADIPOQ*, Sequenom’s MassARRAY EpiTYPER protocol was applied (Sequenom, San Diego, CA, USA). Assays were designed using EpiDesigner (Sequenom). The human *LEP* promoter contains a CpG island that is associated with demethylation during adipocyte differentiation [30]; hence, we aimed to study DNA methylation in this region. Two EpiTYPER assays were designed to cover the *LEP* proximal promoter region, including 57 CpG sites. Due to either low or high mass of the DNA cleavage lengths, in total 39 CpG sites generated measurable DNA methylation data (Fig. 2a). A proximal *ADIPOQ* promoter region has been shown to be sufficient for basal transcriptional activity [31], and additionally, a distal enhancer region has also been shown to affect adiponectin promoter activity [32]. Hence, one assay was designed in the enhancer region and one assay in the proximal promoter, covering in total 10 measurable CpG sites (Fig. 2b). Primer sequences are shown in Additional file 1: Table S1. Due to base-specific cleavage, several CpG sites were analyzed as units, as indicated in Fig. 2.

Genomic DNA (400 ng) from adipose tissue was bisulfite converted using the high-throughput EZ-96 DNA Methylation Kit (ZYMO Research, Orange, CA, USA). Bisulfite-specific primers (Additional file 1: Table S1) were used to generate PCR amplicons. The PCR amplicons were then processed using the MassCleave (hMC) kit. The reverse transcribed cleavage products were dispensed onto a 384 element SpectroCHIP bioarray and using the MassARRAY mass spectrometer (Sequenom), mass spectra were obtained and DNA methylation ratios were analyzed by the EpiTYPER software v.1.0.1 (Sequenom).

### Gene expression

The miRNeasy Mini Kit (Qiagen) was used for total RNA extraction. cDNA synthesis was performed with the QuantiTect Reverse Transcription Kit (Qiagen). Messenger RNA (mRNA) expression of *LEP*, *ADIPOQ* and the reference gene cyclophilin A (*PPIA*) was measured by quantitative real-time PCR (q-PCR) using the ABI PRISM 7900HT Sequence Detection System (Applied Biosystems) and assays-on-design for *LEP* (Hs00174877_m1*, FAM-labelled), *ADIPOQ* (Hs00605917_m1*, FAM-labelled), and *PPIA* (43263116E, VIC-labelled) (all Applied Biosystems). The standard curve principal was applied for gene expression quantification and samples were normalized to *PPIA*. Birth weight and fasting did not affect the expression of *PPIA* (Additional file 2: Figure S1).

### Statistical methodology

Analyses for parametric data were performed using the paired and unpaired Student *t* test and analyses of non-parametrically distributed data were performed using the Wilcoxon test for paired data and the Mann-Whitney *U*-test for unpaired data. Correlation analyses were performed using the Pearson correlation test for parametric data and Spearman rank correlation test for non-parametric data. A mixed effects model was applied to analyze the repeated measurements of p-leptin and p-adiponectin at all four time points of collection under the choice of an unstructured correlation model according to the structure of data, the distribution of the residual variances and the number of parameters involved. In the mixed effects model, the residuals of p-leptin were found to have a non-parametric distribution and log transformation of p-leptin levels was therefore performed. All statistical tests were performed at a significance level of *p* ≤ 0.05, using SAS 9.4 software (SAS Institute Inc., USA).

## Results

### Study subject characteristics

Clinical characteristics of both birth weight groups during the 36-h fasting study have previously been described [27] and are shown in Table 1.

### Influence of fasting on LEP and ADIPOQ DNA methylation in SAT

**Fig. 3:**
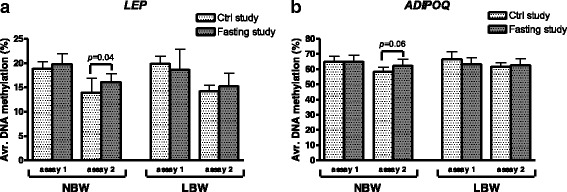
Influence of fasting on DNA methylation of *LEP* (**a**) and *ADIPOQ* (**b**) in SAT from NBW and LBW men. *Avr.* average. *N* = 8 LBW, 8 NBW. Comparisons by paired analyses between control and fasting study. Data are mean ± SD

**Fig. 4:**
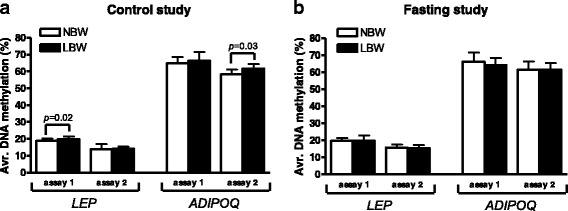
Influence of birth weight on DNA methylation of *LEP* and *ADIPOQ* in SAT. **a** Control study: *Avr.* average. *N* = 8 LBW, 8 NBW. **b** Fasting study: *N* = 20 LBW, 17 NBW. Comparisons by unpaired analyses between NBW and LBW subjects. Data are mean ± SD

The degree of *LEP and ADIPOQ* DNA methylation was positively correlated between the different CpG sites within an assay, in each gene promoter (all *p* ≤ 0.04, results not shown). We therefore performed statistical analysis of the average degree of DNA methylation across all sites within each assay (Figs. 2, 3, and 4) as well as for the individual CpG sites (Additional file 1: Tables S2, S3, S4, and S5).

Among NBW subjects, the average degree of DNA methylation increased significantly with 2.2% in the promoter of *LEP* (assay 2, *p* = 0.04) and borderline significantly with 3.9% in *ADIPOQ* (assay 2, *p* = 0.06), after 36 h fasting compared to the control study (*N* = 8 + 8 NBW, paired analysis) (Fig. 3a, b). In addition, at the individual CpG sites, *LEP* DNA methylation increased ~5% with fasting at sites −51 and −19 (*p* = 0.01, *p* = 0.03), and decreased 2.3% at site −250 (*p* = 0.007) (Additional file 1: Table S2). For *ADIPOQ*, DNA methylation increased 5.4% with fasting at site −327 among the NBW subjects (*p* = 0.007) (Additional file 1: Table S3).

Among LBW subjects, on the contrary, no influence of fasting was observed in either *LEP* or *ADIPOQ* DNA methylation degree (*N* = 8 + 8 LBW, paired analysis) (Fig. 3a, b, Additional file 1: Tables S2 and S3).

### Influence of birth weight on LEP and ADIPOQ DNA methylation in SAT

In the control study, LBW subjects presented 1.8% higher average degree of *LEP* promoter DNA methylation (assay 1, *p* = 0.02) and 3.3% higher average degree of *ADIPOQ* promoter DNA methylation (assay 2, *p* = 0.03) compared to NBW subjects (*N* = 8 LBW versus 8 NBW, unpaired analysis) (Fig. 4a). In addition, two individual *LEP* CpG sites (−441, −393) displayed higher levels of methylation among LBW subjects (Additional file 1: Table S4). There were no differences in average or site-specific DNA methylation of *LEP* assay 2 or *ADIPOQ* assay 1 between NBW and LBW subjects in the control study (Fig. 4a and Additional file 1: Tables S4 and S5).

After 36 h fasting, there were no differences in average degree of *LEP* or *ADIPOQ* DNA methylation between the two birth weight groups (*N* = 20 LBW versus 17 NBW, unpaired analysis) (Fig. 4b). Nevertheless, one *LEP* CpG site (−519) displayed higher DNA methylation in LBW subjects compared to NBW subjects and one *LEP* CpG unit (covering sites −74, −71, −62) displayed lower methylation degree in LBW subjects compared to NBW subjects, after fasting (*p* = 0.02, *p* = 0.05, Additional file 1: Table S4).

### LEP and ADIPOQ gene expression in SAT before and after fasting

**Fig. 5:**
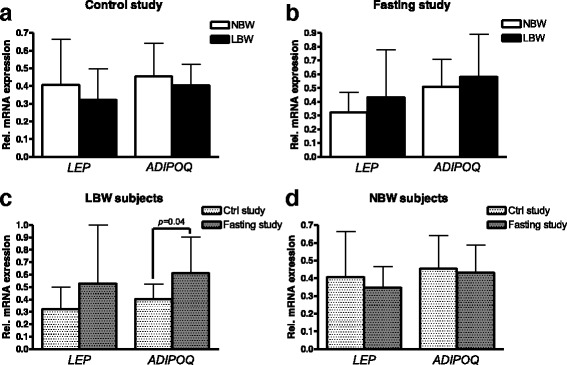
Influence of birth weight (**a**, **b**) and fasting (**c**, **d**) on SAT *LEP* and *ADIPOQ* gene expression levels. *Rel.* relative. **a** Control study: *N* = 8 LBW, 8 NBW. **b** Fasting study: *N* = 20 LBW, 16 NBW, comparisons by unpaired analyses between NBW and LBW subjects. **c**, **d** *N* = 8 LBW, 7 NBW, comparisons by paired analyses between control and fasting study. Data are mean ± SD

Gene expression of *LEP* or *ADIPOQ* did not differ between NBW and LBW subjects in either the control or fasting study (Fig. 5a, b). *ADIPOQ* expression levels increased significantly after fasting among LBW subjects (*p* = 0.04) but not among NBW subjects (Fig. 5c, d). No significant changes in *LEP* gene expression were found with fasting in either of the groups, but especially after fasting a large individual variation was observed in the LBW group (Fig. 5c, d).

### Adipokine plasma profiles before and after fasting

**Fig. 6:**
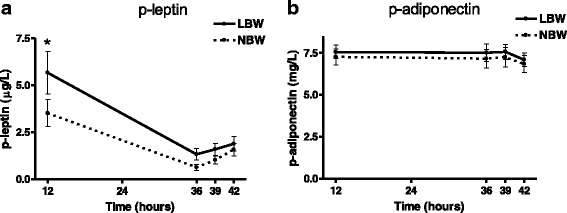
P-leptin (**a**) and p-adiponectin (**b**) levels before and after 36 h fasting in LBW and NBW subjects. *N* = 21 LBW, 18 NBW. Data are means ± SEM. p-leptin levels were significantly higher among LBW subjects after 12 h of fasting (overnight fast) (*p* = 0.05). A threefold decrease of p-leptin levels (*p* < 0.0001) was observed after fasting, with LBW subjects still characterized with non-significant higher p-leptin compared to the NBW subjects. p-leptin levels were also significantly different regarding the slope of the curve between the two groups (*p* = 0.05)

At baseline, after the overnight fast of 12 h, p-leptin levels were significantly higher among LBW subjects compared to the NBW group (*p* = 0.05). After 36 h fasting, both groups exhibited a more than threefold decrease of p-leptin levels (*p* < 0.0001) (Fig. 6a). When performing a mixed effects model of repeated measurements of p-leptin levels during the entire intervention study, the p-leptin effect over time was found to be significantly different between the groups (*p* = 0.01).

No difference was found in p-leptin levels between the groups after either the IVGTT or meal test, when examined by mixed effects models. In addition, these interventions did not appear to have any effect on p-leptin levels, which remained low after the fasting challenge in both groups, though a slight and non-significant increase was observed in both groups (Fig. 6a).

The fasting intervention did not affect p-adiponectin levels in either of the groups, and no differences were found in p-adiponectin levels between the groups at any of the time points during the intervention study (Fig. 6b).

### Associations between SAT LEP and ADIPOQ DNA methylation and mRNA expression

Next, we investigated whether DNA methylation degree of the *LEP* and *ADIPOQ* promoter was associated to gene expression levels in SAT. A significant positive correlation was found between average *LEP* DNA methylation (assay 2) and mRNA expression only in the NBW group (*p* = 0.01) (Table 2). *ADIPOQ* DNA methylation in the proximal promoter (assay 2) was inversely associated with mRNA expression levels in both groups, with a borderline significance when groups were combined (*p* = 0.07) (Table 2).

**Table 2:** Associations between adiposity and *LEP* and *ADIPOQ* SAT DNA methylation and expression after 36 h fasting

	LBW	NBW	Groups combined	LBW	NBW	Groups combined	LBW	NBW	Groups combined
*LEP*	Mean *LEP* DNA methylation assay 1			Mean *LEP* DNA methylation assay 2			P-leptin (μg/L)		
Gene expression	0.23 (0.33)	0.13 (0.62)	0.23 (0.17)	0.08 (0.75)	**0.63 (0.01)**	0.27 (0.10)	0.30 (0.20)	**0.78 (<0.001)**	**0.47 (0.004)**
BMI (kg/m^2^)	0.35 (0.13)	0.06 (0.82)	0.22 (0.19)	0.19 (0.41)	0.22 (0.40)	0.21 (0.21)	**0.47 (0.03)**	**0.68 (0.002)**	**0.52 (<0.001)**
Total BF%	**0.45 (0.05)**	0.18 (0.49)	**0.35 (0.03)**	**0.45 (0.04)**	0.39 (0.12)	**0.42 (0.009)**	**0.42 (0.06)**	**0.83 (<0.001)**	**0.61 (<0.001)**
P-leptin (μg/L)	**0.60 (0.006)**	0.16 (0.53)	**0.49 (0.002)**	0.04 (0.76)	0.27 (0.29)	0.18 (0.28)	–	–	–
*ADIPOQ*	Mean *ADIPOQ* DNA methylation assay 1			Mean *ADIPOQ* DNA methylation assay 2			P-adiponectin (mg/L)		
Gene expression	0.15 (0.52)	−0.23 (0.38)	−0.02 (0.89)	−0.30 (0.19)	−0.24 (0.37)	**−0.31 (0.07)**	0.07 (0.78)	0.15 (0.57)	0.001 (0.99)
BMI (kg/m^2^)	0.12 (0.62)	0.14 (0.58)	0.14 (0.39)	0.12 (0.60)	0.04 (0.87)	0.08 (0.64)	−0.21 (0.35)	**−0.65 (0.003)**	**−0.44 (0.005)**
Total BF%	0.23 (0.33)	**0.69 (0.002)**	**0.44 (0.007)**	0.35 (0.13)	0.21 (0.42)	0.28 (0.10)	−0.17 (0.46)	−0.34 (0.17)	−0.17 (0.30)
P-adiponectin (mg/L)	**−0.47 (0.04)**	−0.17 (0.51)	**−0.32 (0.05)**	−0.10 (0.69)	−0.17 (0.52)	−0.13 (0.44)	–	–	–

Data are given as R coefficient (*p* value). *p* values <0.07 are bold. *N* = 20 LBW and 17 NBW for all data, except for gene expression data: *N* = 20 LBW and 16 NBW

*SAT* subcutaneous adipose tissue, *LBW* low birth weight, *NBW* normal birth weight, *BF* body fat

### Associations between adiposity and SAT LEP and ADIPOQ DNA methylation or plasma levels

Finally, we tested if *LEP* and *ADIPOQ* DNA methylation and/or gene expression in SAT were associated to phenotype including the clinical characteristics of BMI, total BF%, and plasma adipokine levels. *LEP* (assay 1 + 2) and *ADIPOQ* (assay 1) DNA methylation was significantly positively associated with total BF% (*p* < 0.05) but not with BMI, when combining the groups (*n* = 37) (Table 2). When performing correlation analysis by birth weight group, only LBW subjects presented a positive association between *LEP* DNA methylation and total BF% (*p* ≤ 0.05), and only NBW subjects present a positive association between *ADIPOQ* (assay 2) DNA methylation and total BF% (*p* = 0.002).

We further evaluated the effect of adiposity on baseline adipokine plasma levels (after 12 h fasting). In both groups, positive correlations were found between p-leptin levels and BMI or BF%, with the strongest associations observed in the NBW group for all variables (Table 2). BMI and BF% showed significant negative associations to p-adiponectin in the NBW group and in both groups combined (*p ≤* 0.005) (Table 2).

## Discussion

To the best of our knowledge, fasting as an intervention has not previously been investigated with regard to effects on DNA methylation changes, either in animals or in humans. This study shows that the promoter DNA methylation of two metabolically important adipokine genes, *LEP* and *ADIPOQ*, are affected by 36 h fasting in SAT only in NBW subjects and not LBW subjects. Furthermore, we show that LBW subjects have higher baseline methylation degree (after overnight fast) of both gene promoters investigated. However, our results do not support that the promoter *LEP* and *ADIPOQ* DNA methylation degree is directly involved in regulating gene expression under a short-term fasting intervention.

The literature investigating effects of nutritional challenges on DNA methylation in humans is limited and includes studies addressing effects of high-calorie diets [6–9]. We have previously found that LBW subjects, when undergoing a high-fat feeding, exhibit less changeability in muscle tissue DNA methylation than matched NBW controls [33]. Together with our current findings showing that only NBW subjects increase DNA methylation with fasting, it leads us to speculate that LBW subjects may be more inflexible in altering the DNA methylation status when metabolically challenged. The extent to which such metabolic inflexibility in terms of acute regulation of DNA methylation may contribute to an increased risk of metabolic disease in LBW subjects remains uncertain.

The shown changes in DNA methylation among NBW subjects occurred mostly at CpG sites located closer to the transcription start site, which was particularly prominent in the *LEP* promoter. These results suggest that the proximal *LEP* promoter region including the TATA box (−30 to −25) and a transcription factor-binding site for CCAAT-enhancer-binding proteins (C/EBP) (covering CpG −51), which previously was shown to be important for regulation of gene expression in rat adipocytes [34], is more receptive to changes and might be an important region in epigenetic regulation. With respect to the observed significant changes in DNA methylation, it cannot be excluded that short-term diet or fasting-induced methylation changes, in contrast to long-term and more constitutive methylation changes, may represent some beneficial cellular effects as a part of a normal physiological response to diet changes. For example, short-term, fasting-induced methylation (and potentially gene expression) changes of *LEP* could relate to a feedback mechanism to decrease the inhibiting effects of leptin on appetite and stimulate the subject to seek refeeding to compensate for the decreased nutrient supply. Brøns et al. demonstrated that when subjected to a 5-day high-fat diet, LBW subjects in contrast to NBW subjects did not increase p-leptin levels [26]. The lacking p-leptin response to high-fat diet may contribute to a relatively lower induction of satiety in LBW subjects. In the current study, we found a higher baseline level of p-leptin among LBW subjects, which also may affect satiety among the LBW men. Moreover, p-leptin decreased with fasting in both birth weight groups.

Despite the decrease in p-leptin with fasting, which is in agreement with previous findings in fasting humans and animals [35–37], we found no effect of fasting on *LEP* gene expression. Epigenetic influences on gene expression have in several studies been proposed to be involved in the pathogenesis of insulin resistance and T2D [4, 10–12, 38, 39]. Indeed *LEP* gene expression has been shown to be switched on by demethylation of specific CpG sites located in the proximal *LEP* promoter, during the differentiation of adipocytes [30]. Inspired by this study, we recently examined *LEP* DNA methylation in differentiating adipocytes isolated from LBW and NBW subjects, and found an increased *LEP* DNA methylation and a decreased expression in mature adipocytes from LBW subjects compared to NBW subjects, further reinforcing the hypothesis that LBW subjects are less capable of regulating leptin secretion [40]. The decreased secretion of leptin in the differentiated adipocytes is not consistent with the increased baseline levels of p-leptin among the LBW men described in the present work, but could be explained by an intrinsic functional impairment in LBW adipocytes, causing a reduced leptin secretion. This may be compensated by an increased adiposity in vivo resulting in increased p-leptin levels. In our current study, no significant differences were found in BMI and BF%, but indeed this has been observed in previous studies of LBW subjects [6, 23].

Regarding adiponectin, previous studies have consistently reported lower circulating adiponectin levels in LBW subjects [41–43], but this association was not replicated here, possibly due to the younger age and matched study participants included in this study. An increase in *ADIPOQ* gene expression levels was observed after fasting among LBW subjects only. This was unexpected but could relate to unknown compensatory mechanisms associated to the fasting-induced insulin resistance. Only a few studies have previously investigated the effects of fasting on *ADIPOQ* expression and the results are of contradictory nature [44–47]. Nevertheless, we speculate that an increased degree of DNA methylation during fasting may contribute to the fasting-induced insulin resistance.

Recently BMI was reported to be associated with methylation degree in SAT, suggesting that obesity may involve pathological pathways that to some extent are epigenetically regulated [3]. In addition, Houde et al*.* reported that DNA methylation levels of *ADIPOQ* in SAT were positively associated with BMI in severely obese patients [48]. In our study, total BF% was found to be positively associated with both *LEP* and *ADIPOQ* promoter methylation in SAT. This further enforces that changes in SAT DNA methylation patterns are associated with obesity and could be involved in energy homeostasis, but to which degree needs to be further studied. Interestingly, LBW subjects had a higher average degree of DNA methylation in both *LEP* and *ADIPOQ* compared to NBW subjects, in the control study. In the same study cohort we also showed that DNA methylation of the metabolic master regulator *PPARGC1A* was higher in the LBW compared to the NBW subjects after 36 h fasting [27]. These results are in line with a previous finding of increased *LEP* promoter DNA methylation in blood from adult subjects whose mothers were exposed to famine during the peri-conceptional period [49].

In both birth weight groups, p-leptin level was positively associated with BMI and total BF%, corresponding well with the theory and previous studies [14, 15]. Additionally, although a significant negative correlation between plasma adiponectin levels and BMI only was observed among the NBW subjects, negative relations were shown at both body fat parameters in both groups. Still, this could indicate that LBW subjects regulate adiponectin levels differentially and not as directly associated to the amount of body fat, as NBW subjects.

There are limitations to this study, i.e., we use birth weight as a “proxy” for fetal environment. Additionally, we do not have data on the reasons for the low birth weight, e.g., maternal smoking in pregnancy or parental height and cannot exclude possible causes.

The methylation differences that we report here in SAT are slightly smaller than we observed in our previous study of *PPARGC1A* in muscle between LBW and NBW men after fasting (4.5%) [27]. However, overall the magnitude of birth weight on DNA methylation seems to be modest [9]. Furthermore, a 36-h fasting intervention is a quite short time period, compared to a previous 6-month exercise study where larger effects in DNA methylation changes were observed [4]. Still, the magnitude of the methylation differences we observe in the fasting study is similar to the 5-day overfeeding study in both muscle [8] and SAT [25]. Moreover, we are only studying two genes and cannot exclude that other genes may show bigger differences in DNA methylation between the birth weight groups or with fasting. LBW is a risk factor for complex, polygenic diseases including T2D and it is well established that numerous genes with modest effect sizes in methylation changes contribute to this disease [11, 12]. However, we cannot exclude that the DNA methylation measured in our study could be affected by a stochastic variation in methylation, as earlier suggested [50].

## Conclusions

A short-time metabolic fasting challenge induced small but significant changes in DNA methylation of the *ADIPOQ* and *LEP* gene promoters in adipose tissue of young men. The findings support results from a 5-day overfeeding study, indicating that LBW subjects are less flexible in their regulation of DNA methylation degree than NBW subjects when challenged with metabolic interventions. These differential epigenetic patterns present in LBW subjects may contribute to disease development by malfunctions in the metabolically important adipose tissue.

## Additional files

Additional file 1: Table S1. Primers used in amplification of the specific DNA sequences of the *ADIPOQ* and *LEP* promoter regions. **Table S2.** DNA methylation (%) of CpG sites in the *LEP* promoter in adipose tissue. **Table S3.** DNA methylation (%) of CpG sites in the *ADIPOQ* promoter in adipose tissue. **Table S4.** DNA methylation (%) of CpG sites in the *LEP* promoter in adipose tissue. **Table S5.** DNA methylation (%) of CpG sites in the *ADIPOQ* promoter in adipose tissue. (DOCX 34 kb)

Additional file 2: Figure S1. Influence of birth weight (A and B) and fasting (C and D) on SAT *PPIA* gene expression levels as reference gene. A. Control study: *N* = 8 LBW, 8 NBW, B. Fasting study: *N* = 20 LBW, 16 NBW, comparisons by unpaired analyses between NBW and LBW subjects. C and D.: *N* = 8 LBW, 7 NBW, comparisons by paired analyses between control and fasting study. The standard curve principal was applied for gene expression quantification. (DOCX 27 kb)
