# Supplementary material for: 36 h fasting of young men influences adipose tissue DNA methylation of LEP and ADIPOQ in a birth weight-dependent manner
Source: Clin Epigenetics. 2017 Apr 21;9:40. doi: 10.1186/s13148-017-0340-8 (PMC5399392; doi:10.1186/s13148-017-0340-8)
Supplement: Supplementary file 1 — Primers used in amplification of the specific DNA sequences of the ADIPOQ and LEP promoter regions. Table S2. DNA methylation (%) of CpG sites in the LEP promoter in adipose tissue. Table S3. DNA methylation (%) of CpG sites in the ADIPOQ promoter in adipose tissue. Table S4. DNA methylation (%) of CpG sites in the LEP promoter in adipose tissue. Table S5. DNA methylation (%) of CpG sites in the ADIPOQ promoter in adipose tissue. (DOCX 34 kb) [file 13148_2017_340_MOESM1_ESM.docx]

**Additional files**

**Table S1: Primers used in amplification of the specific DNA sequences of the *ADIPOQ* and *LEP* promoter regions:**

| **Primers specific for *ADIPOQ* regions:** |
| --- |
| **Assay 1** (enhancer): Amplicon product: 436bp, coverage 8 CpG sites.  Forward primer: 5’-TTTTGTTTTGGGAAAAAGATTAGTTT-3’  Reverse primer: 5’-AACAAACACCTACAATCCCAACTAC-3’ |
| **Assay 2** (proximal promoter): Amplicon product: 492bp, coverage 4 CpG sites.  Forward primer: 5’-TAGGGTTTAGGTTAGAGAGTGGAGG-3’  Reverse primer: 5’-TTCCCATTAACCAACTCAATAAAAA-3’ |
| **Primers specific for *LEP* promoter regions:** |
| **Assay 1:** Amplicon product: 315bp, coverage 30 CpG sites.  Forward primer: 5’-GTTAGAGAAGGGGTGGGATTTTAG-3’  Reverse primer: 5’-AAATCCTTAATATCCCTCCAAAAACT-3’ |
| **Assay 2:** Amplicon product: 408bp, coverage 27 CpG sites.  Forward primer: 5’-GAAGTATTTTTTTAAGGGGTTGGT-3’  Reverse primer: 5’-TAACCTACCAAAAAAAACCAACAAA-3 |

| **Table S2 DNA methylation (%) of CpG sites in the *LEP* promoter in adipose tissue** | | | | | |  |
| --- | --- | --- | --- | --- | --- | --- |
|  |  | **LBW(8)** |  |  | **NBW(8)** |  |
| **CpG site** | **Control study** | **36hr fasting** | ***p-*value** | **Control study** | **36hr fasting** | ***p-*value** |
| -519 | 12.3±3.3 | 13.9±4.9 | *0.41* | 10.5±2.8 | 11.0±2.3 | *0.60* |
| -507 | 34.1±4.2 | 35.8±7.8 | *0.47* | 33.4±4.6 | 35.1±6.2 | *0.40* |
| (-500, -495) | 40.3±1.8 | 39.1±7.6 | *0.66* | 38.8±3.3 | 40.1±2.6 | *0.38* |
| -467 | 16.6±2.2 | 15.0±3.6 | *0.28* | 16.0±2.4 | 18.0±3.0 | *0.12* |
| (-457, -454, -448) | 11.1±1.6 | 12.5±4.1 | *0.42* | 11.1±3.0 | 12.8±2.3 | *0.23* |
| -441 | 20.6±3.4 | 17.6±5.1 | *0.23* | 17.6±2.1 | 18.9±2.1 | *0.16* |
| -436 | 20.1±4.4 | 19.3±5.2 | *0.57* | 19.9±3.4 | 19.5±4.9 | *0.73* |
| -425 | 30.5±4.4 | 26.3±7.8 | *0.21* | 26.4±3.5 | 29.5±4.3 | *0.18* |
| -399 | 17.5±5.6 | 16.0±7.0 | *0.38* | 17.1±4.1 | 19.1±4.3 | *0.34* |
| -393 | 27.9±5.5 | 19.6±9.5 | *0.09* | 21.0±5.8 | 22.1±6.8 | *0.89* |
| (-379, -374, -372) | 11.6±1.8 | 10.9±4.8 | *0.71* | 10.4±1.3 | 11.6±2.5 | *0.26* |
| (-350, -345) | 13.0±3.1 | 13.6±2.9 | *0.70* | 11.3±1.9 | 13.1±2.9 | *0.16* |
| (-341 - -325) | 18.0±2.4 | 17.4±2.4 | *0.54* | 17.1±2.3 | 19.5±3.4 | *0.18* |
| **Avr Assay 1** | 21.0±1.6 | 19.6±4.4 | *0.42* | 19.2±0.9 | 20.8±2.2 | *0.23* |
| -295 | 16.4±4.7 | 17.0±5.1 | *0.72* | 13.9±4.9 | 16.9±4.1 | *0.08* |
| (-286, -284, -281) | 27.4±1.7 | 27.9±3.2 | *0.62* | 26.8±4.1 | 28.9±1.6 | *0.12* |
| -250 | 9.5±5.2 | 11.3±7.2 | *0.46* | 12.4±6.5 | 10.1±5.9 | ***0.007*** |
| -170 | 12.6±2.1 | 14.1±2.5 | *0.29* | 14.5±2.5 | 14.3±1.7 | *0.78* |
| (-100, -95) | 7.8±1.6 | 8.8±2.8 | *0.32* | 7.1±1.4 | 8.8±1.7 | *0.08* |
| (-74, -71, -62) | 8.1±1.8 | 8.9±1.6 | *0.35* | 8.6±1.8 | 10.1±2.0 | *0.17* |
| -51 | 12.5±3.0 | 13.5±6.5 | *0.57* | 14.1±3.8 | 18.0±3.7 | ***0.01*** |
| -19 | 16.3±5.8 | 17.4±4.1 | *0.67* | 17.8±6.3 | 23.1±5.1 | ***0.03*** |
| +9 | 1.6±1.6 | 2.0±3.7 | *0.81* | 2.6±3.4 | 1.6±2.6 | *0.58* |
| +14 | 29.9±9.2 | 31.1±7.4 | *0.73* | 21.5±12.6 | 29.1±8.9 | *0.20* |
| **Avr Assay 2** | 14.2±1.3 | 15.2±2.7 | *0.40* | 13.9±3.0 | 16.1±1.7 | ***0.04*** |
| Data are mean±SD. Significant differences between control study and 36 hrs fasting in each birth weight group for paired t-tests, at *p*<0.05. | | | | | | |

| **Table S3 DNA methylation (%) of CpG sites in the *ADIPOQ* promoter in adipose tissue** | | | | | | |
| --- | --- | --- | --- | --- | --- | --- |
|  |  | **LBW(8)** |  |  | **NBW(8)** |  |
| **CpG site** | **Control study** | **36hr fasting** | ***p-*value** | **Control study** | **36hr fasting** | ***p-*value** |
| -2685 | 56.8±4.0 | 53.6±7.2 | *0.34* | 56.4±4.6 | 54.6±5.5 | *0.46* |
| -2648 | 63.1±7.0 | 57.8±10.2 | *0.23* | 60.1±4.8 | 58.8±6.6 | *0.58* |
| -2474 | 67.9±6.4 | 63.1±4.9 | *0.10* | 66.3±4.0 | 66.9±4.7 | *0.79* |
| -2442 | 66.3±7.2 | 62.6±4.7 | *0.28* | 63.1±5.7 | 65.1±7.2 | *0.42* |
| (-2436,-2431) | 72.8±4.2 | 71.6±4.3 | *0.61* | 70.6±3.9 | 72.9±5.3 | *0.43* |
| -2419 | 71.9±5.8 | 70.5±5.3 | *0.57* | 72.3±5.7 | 71.5±6.7 | *0.83* |
| **Avr. enhancer** | 66.4±5.2 | 63.2±4.3 | *0.19* | 64.8±3.8 | 65.0±4.1 | *0.93* |
| -415 | 78±4.3 | 76.4±2.9 | *0.45* | 74.1±4.2 | 76±4.9 | *0.29* |
| -327 | 52.8±3.0 | 55.1±6.5 | *0.33* | 48.9±4.3 | 54.3±5.1 | ***0.007*** |
| -112 | 54.1±3.0 | 56.0±7.3 | *0.52* | 51.8±4.1 | 56.3±5.9 | *0.17* |
| **Avr. promoter** | 61.6±2.6 | 62.5±4.4 | *0.64* | 58.3±2.9 | 62.2±4.3 | *0.06* |
| Data are mean±SD. Significant differences between control study and 36 hrs fasting in each birth weight group for paired t-tests, at *p*<0.05. | | | | | | |

| **Table S4 DNA methylation (%) of CpG sites in the *LEP* promoter in adipose tissue** | | | | | | |
| --- | --- | --- | --- | --- | --- | --- |
|  | **Control study** | |  | **36hr fasting** | |  |
| **CpG site** | **LBW (8)** | **NBW (8)** | ***p-*value** | **LBW (20)** | **NBW (17)** | ***p-*value** |
| -519 | 12.3±3.3 | 10.5±2.8 | *0.27* | 14.9±5.8 | 11.5±2.2 | ***0.02*** |
| -507 | 34.1±4.2 | 33.4±4.6 | *0.74* | 38.6±7.9 | 34.4±6.9 | *0.10* |
| (-500, -495) | 40.3±1.8 | 38.8±3.3 | *0.28* | 41.1±6.0 | 39.1±4.1 | *0.25* |
| -467 | 16.6±2.2 | 16.0±2.4 | *0.59* | 16.6±3.4 | 17.5±2.6 | *0.40* |
| (-457, -454, -448) | 11.1±1.6 | 11.1±3.0 | *1* | 12.4±3.3 | 12.8±2.7 | *0.72* |
| -441 | 20.6±3.4 | 17.6±2.1 | ***0.05*** | 19.0±4.2 | 19.9±2.5 | *0.41* |
| -436 | 20.1±4.4 | 19.9±3.4 | *0.90* | 20.3±4.4 | 19.8±3.7 | *0.75* |
| -425 | 30.5±4.4 | 26.4±3.5 | *0.06* | 26.8±5.6 | 27.5±4.2 | *0.66* |
| -399 | 17.5±5.6 | 17.1±4.1 | *0.88* | 16.8±7.1 | 18.2±4.1 | *0.44* |
| -393 | 27.9±5.5 | 21.0±5.8 | ***0.03*** | 21.4±7.8 | 23.2±6.5 | *0.45* |
| (-379, -374, -372) | 11.6±1.8 | 10.4±1.3 | *0.13* | 11.6±3.3 | 11.8±2.2 | *0.82* |
| (-350, -345) | 13.0±3.1 | 11.3±1.9 | *0.20* | 13.6±2.3 | 12.8±2.4 | *0.29* |
| (-341 - -325) | 18.0±2.4 | 17.1±2.3 | *0.47* | 17.6±2.0 | 19.2±3.1 | *0.06* |
| **Avr Assay 1** | 21.0±1.6 | 19.2±0.9 | ***0.02*** | 20.8±4.9 | 20.6±3.6 | *0.41* |
| -295 | 16.4±4.7 | 13.9±4.9 | *0.31* | 17.0±4.2 | 17.0±4.8 | *1* |
| (-286, -284, -281) | 27.4±1.7 | 26.8±4.1 | *0.70* | 28.8±3.3 | 28.2±2.2 | *0.55* |
| -250 | 9.5±5.2 | 12.4±6.5 | *0.35* | 12.8±6.8 | 9.9±4.8 | *0.16* |
| -170 | 12.6±2.1 | 14.5±2.5 | *0.13* | 13.7±2.2 | 13.6±2.0 | *1* |
| (-100, -95) | 7.8±1.6 | 7.1±1.4 | *0.41* | 8.5±2.0 | 8.7±1.9 | *0.75* |
| (-74, -71, -62) | 8.1±1.8 | 8.6±1.8 | *0.59* | 8.8±1.7 | 9.9±1.6 | ***0.05*** |
| -51 | 12.5±3.0 | 14.1±3.8 | *0.36* | 14.4±4.6 | 16.4±3.8 | *0.15* |
| -19 | 16.3±5.8 | 17.8±6.3 | *0.63* | 18.5±3.3 | 20.7±5.2 | *0.12* |
| +9 | 1.6±1.6 | 2.6±3.4 | *0.47* | 1.9±3.2 | 2.4±4.5 | *0.69* |
| +14 | 29.9±9.2 | 21.5±12.6 | *0.15* | 30.3±6.4 | 30.7±8.7 | *0.86* |
| **Avr Assay 2** | 14.2±1.3 | 13.9±3.0 | *0.81* | 15.5±1.9 | 15.8±1.8 | *0.60* |
| Data are mean±SD. Significant differences between the birth weight groups for unpaired t-tests between control study and 36 hrs fasting, at *p*<0.05. | | | | | | |

| **Table S5 DNA methylation (%) of CpG sites in the *ADIPOQ* promoter in adipose tissue** | | | | | | |
| --- | --- | --- | --- | --- | --- | --- |
|  | **Control study** | |  | **36hr fasting** | |  |
| **CpG site** | **LBW (7)** | **NBW (6)** | ***p-*value** | **LBW (20)** | **NBW (17)** | ***p-*value** |
| -2685 | 56.8±4.0 | 56.4±4.6 | *0.86* | 55.2±6.0 | 57.0±5.9 | 0.37 |
| -2648 | 63.1±7.0 | 60.1±4.8 | *0.34* | 59.8±7.5 | 61.8±7.0 | 0.41 |
| -2474 | 67.9±6.4 | 66.3±4.0 | *0.55* | 64.6±4.4 | 67.5±5.0 | 0.07 |
| -2442 | 66.3±7.2 | 63.1±5.7 | *0.35* | 63.2±4.7 | 65.9±7.6 | 0.18 |
| -2436,-2431 | 72.8±4.2 | 70.6±3.9 | *0.31* | 71.5±3.9 | 73.1±5.7 | 0.34 |
| -2419 | 71.9±5.8 | 72.3±5.7 | *0.90* | 71.1±5.0 | 72.2±6.5 | 0.54 |
| **Avr. enhancer** | 66.4±5.2 | 64.8±3.8 | *0.48* | 64.2±4.2 | 66.2±5.3 | 0.20 |
| -415 | 78±4.3 | 74.1±4.2 | *0.09* | 77.2±3.4 | 77.0±3.9 | 0.90 |
| -327 | 52.8±3.0 | 48.9±4.3 | *0.06* | 53.5±5.5 | 52.5±6.2 | 0.64 |
| -112 | 54.1±3.0 | 51.8±4.1 | *0.21* | 54.4±6.5 | 55.2±6.9 | 0.71 |
| **Avr. promoter** | 61.6±2.6 | 58.3±2.9 | ***0.03*** | 61.7±3.9 | 61.6±4.7 | 0.95 |
| Data are mean±SD. Significant differences between the birth weight groups for unpaired t-tests between control study and 36 hrs fasting, at *p*<0.05. | | | | | | |
