# Supplementary material for: 36 h fasting of young men influences adipose tissue DNA methylation of LEP and ADIPOQ in a birth weight-dependent manner
Source: Clin Epigenetics. 2017 Apr 21;9:40. doi: 10.1186/s13148-017-0340-8 (PMC5399392; doi:10.1186/s13148-017-0340-8)
Supplement: Supplementary file 2 — Influence of birth weight (A and B) and fasting (C and D) on SAT PPIA gene expression levels as reference gene. A. Control study: N = 8 LBW, 8 NBW, B. Fasting study: N = 20 LBW, 16 NBW, comparisons by unpaired analyses between NBW and LBW subjects. C and D.: N = 8 LBW, 7 NBW, comparisons by paired analyses between control and fasting study. The standard curve principal was applied for gene expression quantification. (DOCX 27 kb) [file 13148_2017_340_MOESM2_ESM.docx]

**Figure S1: Influence of birth weight (A and B) and fasting (C and D) on SAT *PPIA* gene expression levels as reference gene.** **A.** Control study: *N*=8 LBW, 8 NBW, **B.** Fasting study: *N*=20 LBW, 16 NBW, comparisons by unpaired analyses between NBW and LBW subjects. **C and D.**: *N*=8 LBW, 7 NBW, comparisons by paired analyses between control and fasting study. The standard curve principal was applied for gene expression quantification.
